# A Retroperitoneal Leiomyosarcoma Presenting as an Adrenal Incidentaloma in a Subject on Warfarin

**DOI:** 10.1155/2015/830814

**Published:** 2015-04-29

**Authors:** Ishrat N. Khan, Mohamed A. Adlan, Michael J. Stechman, Lakdasa D. Premawardhana

**Affiliations:** ^1^Section of Endocrinology, Ysbyty Ystrad Fawr, Ystrad Fawr Way, Hengoed, Caerphilly CF82 7EP, UK; ^2^Department of Endocrine Surgery, University Hospital of Wales, Heath Park, Cardiff CF14 4XN, UK; ^3^Department of Endocrinology, University Hospital of Wales, Heath Park, Cardiff CF14 4XN, UK

## Abstract

Adrenal incidentalomas (AIs) are mostly benign and nonsecretory. Management algorithms lack sensitivity when assessing malignant potential, although functional status is easier to assess. We present a subject whose AI was a retroperitoneal leiomyosarcoma (RL). *Case Presentation*. A woman on warfarin with SLE and the antiphospholipid syndrome, presented with left loin pain. She was normotensive and clinically normal. Ultrasound scans demonstrated left kidney scarring, but CT scans revealed an AI. MRI scans later confirmed the AI without significant fat and no interval growth. Cortisol after 1 mg dexamethasone, urinary free cortisol and catecholamines, plasma aldosterone renin ratio, and 17-hydroxyprogesterone were within the reference range. Initially, adrenal haemorrhage was diagnosed because of warfarin therapy and the acute presentation. However, she underwent adrenalectomy because of interval growth of the AI. Histology confirmed an RL. The patient received adjuvant radiotherapy. *Discussion*. Our subject presented with an NSAI. However, we highlight the following: (a) the diagnosis of adrenal haemorrhage in this anticoagulated woman was revised because of interval growth; (b) the tumour, an RL, was relatively small at diagnosis; (c) this subject has survived well over 60 months despite an RL perhaps because of her acute presentation and early diagnosis of a small localised tumour.

## 1. Introduction

Adrenal incidentalomas (AIs) are a modern phenomenon [1] and several algorithms are available for their management [2–4]. Clinicians managing an AI have two decisions to make: (a) is it secretory and (b) is it malignant? Surgical intervention is indicated if clinical, laboratory, or radiological features of either or both are present. The functional status of AI is relatively straightforward to assess. If nonsecretory adrenal incidentalomas (NSAI) do not fulfil size and radiological criteria [5], a “wait and see” policy with interval scanning is recommended. Such a policy is not fail safe and may occasionally lead to undesirable outcomes. This is particularly so when the patient has comorbidities and is on drugs which may contribute to diagnostic confusion.

AIs are usually benign and nonsecretory [2]. The current guidelines for surgery for NSAI are based on tumour size and appearance on CT scanning (MRI or PET if used) (Table 1). For NSAI that do not fulfil size criteria at presentation, features such as increasing size and an unfavourable imaging “phenotype” may indicate the need for surgery [6]. The lipid content of benign NSAI is high and CT “attenuation” values are significantly lower than that for adrenal carcinomas, metastases, or pheochromocytomas [7]. Noncontrasted CT attenuation values of <10 HU are typical of benign lesions, but lipid poor benign NSAI (10–40% of benign adenomas) may occasionally show higher values. Contrast washout tends to be lower (<40%) in carcinomas compared to benign adenomas. Furthermore, malignant lesions may demonstrate local invasion, necrosis, heterogeneous appearances, irregular borders, and regional lymphadenopathy [8]. However, neither tumour size nor the imaging “phenotype” is sensitive enough to differentiate between benign and malignant lesions in every patient [9]. These appearances may be distorted particularly in subjects who have had a haemorrhage into the lesion, either spontaneously or complicating anticoagulant therapy. Rarely NSAIs are malignant, primary or metastatic (renal, lung, gastrointestinal, and bladder). Leiomyosarcomas presenting as NSAI are very uncommon.

We present a subject who had a very rare high-grade retroperitoneal leiomyosarcoma (RL) presenting as an NSAI. She also had the antiphospholipid syndrome and was on warfarin therapy.

## 2. Case Presentation

**Figure 1:**
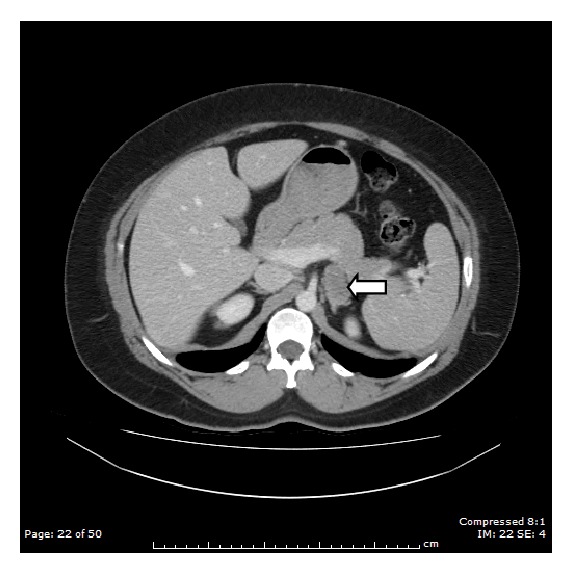
A CT scan of the abdomen showing a well encapsulated “adrenal” mass (solid white arrow).

A 40-year-old woman with SLE and the antiphospholipid syndrome presented acutely with sudden onset left sided loin pain. She was on life-long warfarin therapy for multiple DVTs and pulmonary emboli and hydroxychloroquine. Ultrasound scans showed a 10 mm focal area in the left kidney due to scarring or a small angiomyolipoma. A CT scan several months later showed a 2.7 × 3.9 cm left adrenal incidentaloma (Figure 1), and she was referred to the endocrine clinic.

On examination, she was normotensive and had no clinical signs of endocrinopathy. Investigations including cortisol after 1 mg dexamethasone suppression overnight, urinary free cortisol (twice), urinary catecholamines (twice), aldosterone renin ratio, 17-hydroxyprogesterone, and chromogranins were all within the reference range. MRI scans showed a solid left adrenal lesion measuring 2.4 × 4 cm. without significant fat and no interval growth. However, in view of the phenotype and size of the adrenal mass she was referred to the endocrine surgeons. Following a review of her history of loin pain, and imaging, it was decided that she had a likely benign NSAI (uniform soft tissue signal intensity, well-defined margins, no local invasion, appropriate size, and no interval growth over 3 months), who may have developed acute symptoms as a result of haemorrhage into the lesion. Therefore a conservative approach was adopted with scans to be repeated in 3–6 months. Although the size of the lesion initially remained stable, imaging one year later revealed that it had increased in size to 3 × 5.4 cm. The patient therefore had left laparoscopic adrenalectomy. At surgery, there was no macroscopic invasion of adjacent structures and the lesion was excised with periadrenal fat.

**Figure 2:**
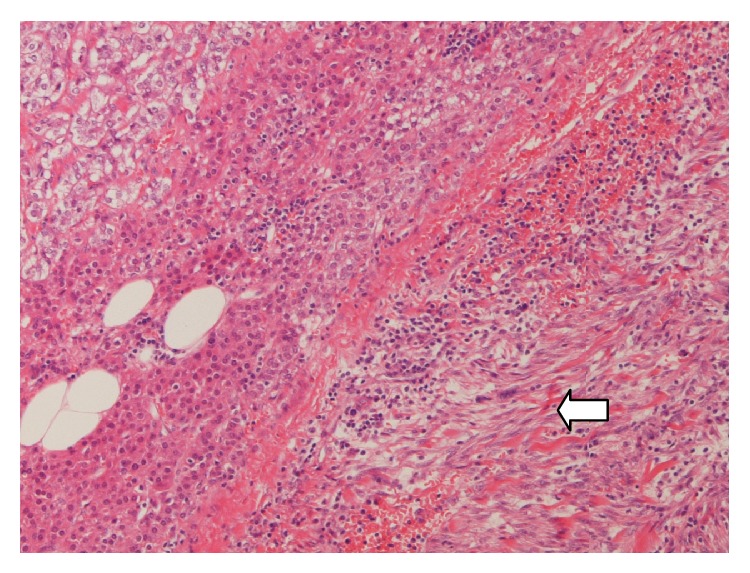
Microscopic appearances of the leiomyosarcoma on the right (solid white arrow) and normal adrenal gland on the left (haematoxylin and eosin staining). The cells were strongly positive for smooth muscle actin and desmin on immunostaining (not shown).

Histology revealed a nonencapsulated high-grade leiomyosarcoma thought to have arisen from connective tissue but separate from the adrenal gland, that is, a retroperitoneal leiomyosarcoma (RL). There were atypical pleomorphic spindle cells forming a herringbone pattern in areas (Figure 2). These cells had cigar shaped nuclei with some multinucleated forms. Immunostaining revealed strong expression of smooth muscle actin and desmin. There was no AE1/AE3, S100, CD117, CD34, or CD99 expression. Proliferation fraction on Ki67 immunostaining was approximately 30%. Staging CT confirmed only localised disease. She had radiotherapy as recommended by the Sarcoma MDT.

## 3. Discussion

We have described a 40-year-old woman who presented with what appeared to be a benign NSAI. It was later confirmed that the “NSAI” was actually an RL and separate from the adrenal gland. As far as we are aware, this is the first report of an RL presenting as an AI. Retroperitoneal sarcomas are rare and RL even more so [10]. They are largely asymptomatic and therefore grow to a large size before presentation—50% are more than 15 cm in diameter. They have a very poor prognosis. Their large size and spread to surrounding structures at presentation, and technical difficulties in surgical removal, contribute to this poor prognosis [11]. RL usually arises from the walls of retroperitoneal veins most often from the inferior vena cava and shows no gender disparity. Radical surgery followed by radiotherapy and/or chemotherapy is recommended.

The management of our subject was complicated by her drug therapy and the nonspecific appearances on imaging. It is noteworthy that her initial abdominal ultrasound scan was reported to show renal scarring or an angiomyolipoma of the kidney, both benign entities. An AI was only identified later on CT scanning. Endocrine assessment at this stage confirmed it to be an NSAI, but despite a lack of interval growth, it was felt, on size criteria and a paucity of fat in the lesion on imaging, that surgery should be considered [12]. The combination of her acute presentation with loin pain on the same side as the lesion, the absence of worrying radiological appearances at that stage, and the inherent risk of surgery in an anticoagulated woman with the antiphospholipid syndrome pushed the balance of risks away from immediate surgery. However, interval growth of the mass during follow-up required a reevaluation of the diagnosis so that urgent resection was pursued.

In conclusion, we would like to highlight the following.

- The diagnosis of an adrenal bleed in this anticoagulated woman was revised because of interval growth of the mass.
- This growth was only detectable 1 year after first evaluation. Lack of early interval growth does not exclude a malignant lesion in every subject; we support repeating initial negative scans as recommended in recent algorithms [7].
- Surgery should be considered for 4 cm diameter AIs showing an appropriate noncontrast CT phenotype.
- While primary adrenal leiomyosarcomas are very rare (only 20 cases reported so far), RL may very rarely present as an NSAI.
- This tumour was relatively small compared to typical RLs described in the literature—50% more than 15 cm in diameter. This was most likely because of her acute presentation with loin pain, which prompted imaging which led to an earlier diagnosis.
- This subject has survived for well over 60 months despite a high-grade malignant RL perhaps because of early detection and a small localised tumour, resulting from her acute presentation.

## Figures and Tables

**Table 1:** Adrenal imaging modalities and their utility.

Scan type	Diagnostic characteristics	Comments
Contrast enhanced ultrasound	Arterial enhancement and rapid washout seen in nonadenomatous lesions	Not commonly used

Unenhanced CT scan	(a) Presence of macroscopic fat seen in myelolipoma (b) Attenuation of less than 10 Hounsfield units (HU) seen in benign adrenal adenomas	Commonly used scan mode but 30–40% benign lesions are lipid poor

Multiphase CT	(a) Rapid washout, benign lesions (b) Slower washout, malignant lesions	Useful when unenhanced CT is equivocal

MRI	Chemical shift imaging is helpful in differentiating between lipid poor adenomas and malignant adrenal lesions	Helpful when CT is contraindicated or washout values are equivocal

Positron emission tomography (PET) and PET-CT	Differentiate benign from malignant lesions with high sensitivity and specificity	Combine HU measurement with functional activity
